# Probabilistic Modeling of Multicamera Interference for Time-of-Flight Sensors

**DOI:** 10.3390/s23198047

**Published:** 2023-09-23

**Authors:** Bryan Rodriguez, Xinxiang Zhang, Dinesh Rajan

**Affiliations:** Department of Electrical and Computer Engineering, Lyle School of Engineering, Southern Methodist University, Dallas, TX 75205, USA; xinxiang@mail.smu.edu (X.Z.); rajand@lyle.smu.edu (D.R.)

**Keywords:** 3D image processing, multicamera interference, depth maps, time-of-flight sensors

## Abstract

The behavior of multicamera interference in 3D images (e.g., depth maps), which is based on infrared (IR) light, is not well understood. In 3D images, when multicamera interference is present, there is an increase in the amount of zero-value pixels, resulting in a loss of depth information. In this work, we demonstrate a framework for synthetically generating direct and indirect multicamera interference using a combination of a probabilistic model and ray tracing. Our mathematical model predicts the locations and probabilities of zero-value pixels in depth maps that contain multicamera interference. Our model accurately predicts where depth information may be lost in a depth map when multicamera interference is present. We compare the proposed synthetic 3D interference images with controlled 3D interference images captured in our laboratory. The proposed framework achieves an average root mean square error (RMSE) of 0.0625, an average peak signal-to-noise ratio (PSNR) of 24.1277 dB, and an average structural similarity index measure (SSIM) of 0.9007 for predicting direct multicamera interference, and an average RMSE of 0.0312, an average PSNR of 26.2280 dB, and an average SSIM of 0.9064 for predicting indirect multicamera interference. The proposed framework can be used to develop and test interference mitigation techniques that will be crucial for the successful proliferation of these devices.

## 1. Introduction

In the last decade, there has been in increase in the types of applications where 3D sensors are used, such as 3D scanning [1,2,3,4], drone positioning [5,6,7], robotics [8,9,10], and logistics [11,12,13]. In particular, there has been a rapid growth in the commoditization of 3D sensors and their integration into mobile smartphone devices. In such a situation, there is an increasing likelihood of these sensors causing interference to each other during regular operation in many practical settings, such as multiple cameras imaging the same object or subject. There are only a limited number of previous works that quantify the behavior of multicamera interference in 3D images (e.g., depth maps) that is captured by a time-of-flight (ToF) sensor using infrared (IR) light [14,15,16,17,18,19]. Existing works describe the behavior of multicamera interference using a simple mathematical model based on a summation of modulated light signals that corresponds with the IR light that is emitted from each ToF sensor [14,15,16,17]. These simple models characterize how the accuracy of a depth measurement at a given pixel is affected by IR light that is contributed by other ToF sensors. These models, however, do not predict the locations of zero-value pixels within a depth map and do not consider the impact of the physical locations of the ToF sensors with respect to each other. For example, existing models do not describe the effect that one ToF sensor has on another ToF sensor based on an offset (e.g., a horizontal and/or vertical offset) between the ToF sensors within the same image plane, or an offset (i.e., parallax) between the image planes of the ToF sensors. The physical locations of other ToF sensors have a significant impact on the amount of multicamera interference that is present. For example, ToF sensors that are physically closer to each other or facing towards each other will experience more multicamera interference than other ToF sensor configurations.

In this work, we develop a framework for synthetically generating multicamera interference within a depth map that mirrors real multicamera interference that is observed by a primary ToF sensor in the presence of an interfering secondary ToF sensor. In this framework, we introduce a probabilistic model that predicts the behavior of multicamera interference in depth maps based on the relative physical locations of the ToF sensors and any potential reflecting surface. Generally, when multicamera interference is present, there is an increase in the amount of zero-value pixels in the depth map captured by the primary ToF sensor. A zero-value pixel is an invalid pixel that carries no depth information. Hence, an increase in the amount of zero-value pixels in a depth map corresponds to an increase in the depth information that is lost. Our experiments show that non-zero-value pixels (i.e., pixels with real depth values) experience negligible amounts of error, generally less than 3 mm at operating distances of 1 m, due to multicamera interference. Thus, the focus of this work is to predict the behavior of zero-value pixels in a depth map when multicamera interference is present.

The ToF sensors considered in this work rely primarily on frequency hopping for eliminating multicamera interference. This frequency-hopping approach generally operates by only transmitting IR light at different frequencies that vary over time [20,21,22,23]. Due to hardware constraints, a limited number of frequencies are typically available for a device to use. As the number of devices imaging a scene increases, the likelihood of the same frequencies being used simultaneously by more than one device increases. When multiple devices transmit IR light at the same frequency, this situation results in multicamera interference [24]. As such, existing frequency-hopping techniques alone are insufficient for eliminating multicamera interference. Our framework and probabilistic model can help to improve existing approaches for eliminating multicamera interference, since they can be used to predict the locations and probabilities of zero-value pixels that are present in a depth map when multicamera interference is present and compensate for loss in depth information.

This work contributes to the state of the art in the following ways: (1) Developing a framework for synthetically generating (a) direct multicamera interference in depth maps using a probabilistic model and (b) indirect multicamera interference in depth maps using ray tracing and the probabilistic model. The synthetically generated multicamera interference accurately mimics what is observed by a primary ToF sensor in the presence of an interfering secondary ToF sensor; (2) developing a mathematical model that predicts the locations and probabilities of invalid or zero-value pixels being present in depth maps that contain multicamera interference. The mathematical model uses a probabilistic approach to predict the probability of a pixel being a zero-value pixel when direct multicamera interference is present. Direct interference occurs when an IR light source, for example from an interfering secondary ToF sensor, has a line-of-sight path with a primary ToF sensor. In this model, probabilities are determined using a sigmoid function and the position of a given pixel in an image sensor with respect to a pixel corresponding with a center of an IR light source; (3) developing a ray-tracing approach that uses the geometry of a scene in conjunction with the probabilistic model for predicting zero-value pixels in direct multicamera interference to predict zero-value pixels associated with indirect multicamera interference. Indirect multicamera interference occurs when the IR light from an interfering IR light source is reflected off of a surface before being observed by the primary ToF sensor. This ray-tracing approach virtually projects and diffuses the IR light from an IR light source onto surfaces in a scene to predict the locations and probabilities of zero-value pixels that are associated with indirect interference; and (4) quantifying the performance of the synthetic depth interference images with controlled 3D interference images captured in our laboratory. The proposed framework achieves an average root-mean-square-error (RMSE) of 0.0625, an average peak signal-to-noise ratio (PSNR) of 24.1277 dB, and an average structural similarity index measure (SSIM) of 0.9007 for predicting the locations and probabilities of zero-value pixels for direct multicamera interference, and an average RMSE of 0.0312, an average PSNR of 26.2280 dB, and an average SSIM of 0.9064 for predicting the locations and probabilities of zero-value pixels for indirect multicamera interference.

This work uses Kinect V2 sensors as both a primary ToF sensor and an interfering secondary ToF sensor for experiments due to its popularity and widespread use in research and engineering applications. Since other ToF sensors operate using the same principles, which involves emitting and capturing reflected IR light, our framework for synthetically generating multicamera interference within a depth map can therefore be generally applied to other types of ToF sensors.

This paper is organized as follows. Section 2 discusses the proposed method to synthetically generate multicamera interference within a depth map. Section 3 discusses the experimental setup and the numerical results. Section 4 provides concluding remarks.

## 2. Methodology

**Figure 1:**
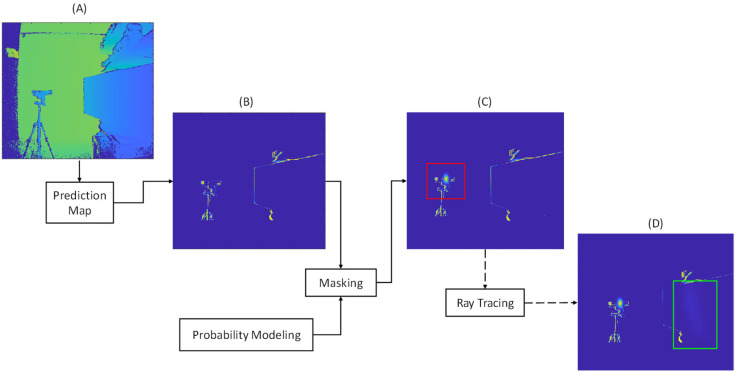
Process for generating synthetic multicamera interference. (**A**) Input depth map from a ToF sensor without multicamera interference; (**B**) Initial prediction map for the input depth map from ToF sensor without multicamera interference; (**C**) Prediction map after applying synthetic direct interference. The added synthetic direct interference is shown within the red bounding box; (**D**) Prediction map after applying synthetic indirect interference. The added synthetic indirect interference is shown within the green bounding box.

Multicamera interference primarily appears in depth maps as an increase in the number of zero-value or invalid pixels within a scene. ToF sensors operate by determining depth values based on the amount of time it takes for an IR light that is emitted from the ToF sensor to return to the ToF sensor, after reflecting off of a surface within a scene. When a different IR source, such as another ToF sensor, emits IR light onto the same scene as that being imaged by a primary ToF sensor, it causes the primary ToF sensor to receive additional and unexpected IR light from the interference source. The additional emitted IR light may appear as direct interference and/or indirect interference in the depth maps that are captured by the primary ToF sensor. The additional IR light results in a loss of depth information that does not accurately represent the object in the scene. In this work, we propose an approach for synthetically generating the presence of multicamera interference within a depth map. The resulting synthetically generated depth map mimics the appearance and behavior of a depth map that would be observed if an interference source was emitting IR light onto a scene while a ToF sensor is collecting data from the scene. Figure 1 provides an overview of our methodology, and the frequently used notations are provided in Table 1.

The key steps to the synthetic multicamera interference generation methodology are as follows. Consider a primary ToF sensor that is used to capture a depth map of a scene without any interference from an interference source. The captured depth map may be formed from a composite of one or more depth map images that are captured over some period of time to improve the signal-to-noise ratio. An initial prediction map is then generated based on the interference-free depth map. In the initial prediction map, each pixel is associated with a probability that corresponds with how often a pixel had a zero-value within the series of collected depth map images that are collected over the period of time. This initial prediction map is provided as an input to the synthetic data generator. Now also consider an interfering secondary ToF sensor that is located in the field-of-view (FOV) of the primary ToF sensor and has its orientation such that a portion of its emitted light is reflected off of the scene onto the primary ToF sensor. This generalized configuration captures the possibility that (1) the interfering secondary ToF sensor does not lie in the primary ToF sensor’s FOV but still causes interference and (2) the interfering secondary ToF sensor lies in the primary ToF sensor’s FOV but causes only direct interference and no reflected interference. Positional information about the primary ToF sensor, the interfering secondary ToF sensor, and the reflective surface (which for simplicity is modeled as a 2D plane with a certain orientation) in the scene is given.

The primary objective of this work is to compute an accurate interference image that integrates both the direct and indirect interference components. The proposed method uses probabilistic modeling with the positional information for the interference source to generate a probability map. The probability map associates each pixel within a depth map with a probability of the pixel being a zero-value pixel as a result of multicamera interference. In the case of direct interference, we map the probabilities from the probability map onto an initial prediction map for the depth map of the scene without multicamera interference. Optionally, if positional information is known for other objects or surfaces in the scene, then ray tracing is used to project the probability map to the other objects and surfaces in the scene to generate indirect interference within the initial prediction map for the depth map of the scene without multicamera interference. This process is discussed in detail in the following section.

### 2.1. Multicamera Interference

**Figure 2:**
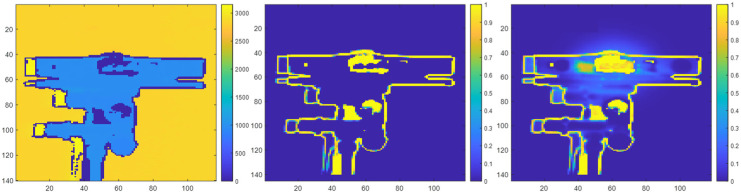
Depth map of an interfering secondary ToF sensor without multicamera interference in millimeter units (**left**). Prediction map of the interfering secondary ToF sensor without multicamera interference (**middle**) and with real direct multicamera interference (**right**).

**Figure 3:**
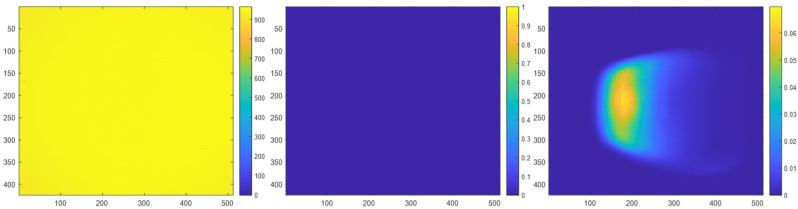
Depth map of a planar surface without multicamera interference in millimeter units (**left**). Prediction map of the planar surface without multicamera interference (**middle**) and with real indirect multicamera interference (**right**).

Multicamera interference can manifest as either direct multicamera interference or indirect multicamera interference within a depth map. Direct multicamera interference occurs when a primary ToF sensor has line of sight to the IR light from an interfering IR light source (e.g., an interfering secondary ToF sensor). An example of real direct multicamera interference is shown in Figure 2. In this example, the IR light source is located at a central portion of the FOV of an interfering secondary ToF sensor. Indirect interference occurs when the IR light from an interfering IR light source is reflected off of a surface before being observed by the primary ToF sensor. An example of real indirect multicamera interference is shown in Figure 3. In this example, IR light from the interfering secondary ToF sensor is being reflected off of a planar surface (e.g., a wall) before being observed by the primary ToF sensor.

**Figure 4:**
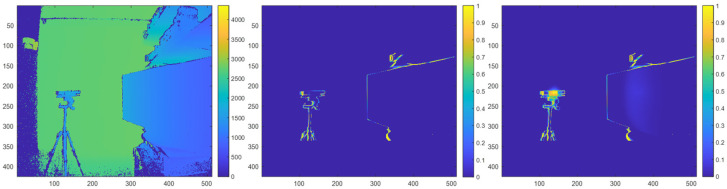
Depth map of a scene without multicamera interference in millimeter units (**left**). Prediction map of the scene without multicamera interference (**middle**) and with real combined direct and indirect multicamera interference (**right**).

In some circumstances, both direct multicamera interference and indirect multicamera interference may be present within a depth map. This situation occurs when the primary ToF sensor has a line of sight with an IR light source and a portion of the IR light from the IR light source is reflected off of a surface before being observed by the primary ToF sensor. An example of real combined direct and indirect multicamera interference is shown in Figure 4. In this example, the primary ToF sensor has line of sight with the IR light source of an interfering secondary ToF sensor. The primary ToF sensor is also able to view a portion of the IR light that is reflected off of a planar target surface.

In the following prediction maps, the probabilities of pixels being a zero-value pixel when multicamera interference is present are normalized based on how often a pixel had a zero-value within a series of depth maps that are collected over a period of time. For example, a pixel that had a zero-value for the entire data sample is associated with a probability of 100% or a value of 1; a pixel that had a zero-value for half of the data samples is associated with a probability of 50% or a value of 0.5. The color bar adjacent to each figure below indicates the mapping between a pixel color and its corresponding probability value. As such, the color of a pixel is used to indicate the probability that is associated with the pixel. A brighter color (e.g., yellow) pixel indicates the pixel is associated with a higher probability of being a zero-value pixel when a multicamera is present, whereas a darker color (e.g., dark blue) pixel indicates the pixel is associated with a lower probability of being a zero-value pixel when multicamera interference is present.

Note that in the figures without multicamera interference, zero-value pixels are still present at the depth discontinuities (e.g., edges) in both the depth maps and their corresponding probability maps due to object boundary ambiguity [25].

### 2.2. Probability Model

In the case of direct interference, a probability map is used to associate each pixel of a depth map with a probability that a pixel is a zero-value pixel when direct multicamera interference is present. 

Let ***D*** ∈ ℝ*^M^*^×*N*^ represent the depth maps obtained from a primary ToF sensor with the dimensions M × N. Each pixel within the depth map is identified using a 2D index $\left(x,y\right)$. The prediction map that corresponds with the depth maps from the primary ToF sensor is also a 2D matrix, ***I*** ∈ ℝ*^M^*^×*N*^, with the same dimensions M × N. Each pixel in the prediction map corresponds with a pixel in the depth map. In the prediction map, each pixel $\left(x,y\right)\;$is associated with an initial probability, $I\left(x,y\right)$, of the pixel being a zero-value pixel. As noted before, this initial probability is determined based on how often a pixel had a zero-value within a series of depth maps that are collected over a period of time.

The probability map is also a 2D matrix, ***P*** ∈ ℝ*^M^*^×*N*^, that is generated with the same dimensions M × N. Each pixel in the probability map corresponds with a pixel in the depth map. Each pixel in the probability map is assigned a predicted probability, $P\left(x,y\right)$, that the pixel is a zero-value pixel when direct multicamera interference is present.

**Figure 5:**
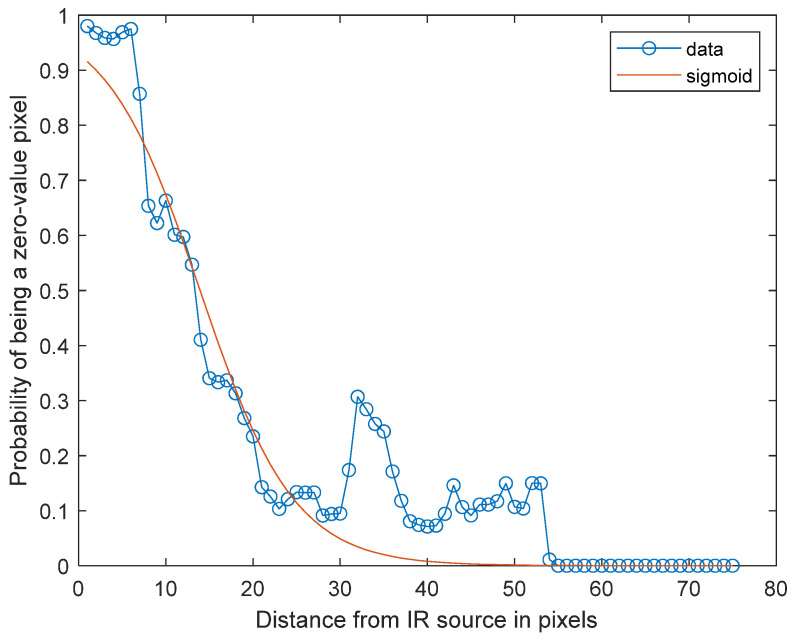
Sigmoid function fit to the zero-value pixel probabilities along a radius projecting away from an IR light.

Based on our experiments, the probability of a zero-value pixel being present for direct multicamera interference is highest at the center of an IR light source and then gradually tapers off as the distance between a pixel and the center of the IR light source increases. Based on this observed behavior, the sigmoid function was chosen as the basis for our probability map to mimic the behavior of direct interference. Figure 5 shows an example of a sigmoid function overlaid with the probabilities of a pixel being a zero-value pixel as a function of the distance between a pixel and the center of the IR light source. While there are numerous other functional choices that can be used, we selected the sigmoid function for the relatively small number of tunable parameters that need to be defined and the high level of accuracy that it offers, as will become evident in the numerical results.

The sigmoid function, S(x,y), is defined as follows:

(1) $$S\left(x,y\right)=\frac{A}{1+e^{-B*\left(d-C\right)}}$$

where d is the Euclidian distance between pixel (x,y) and the center pixel $\left(x_{c},y_{c}\right)$ of an IR light source, S(x,y) is the probability of the pixel (x,y) being a zero-value pixel, A is the peak probability value for the sigmoid function, B is the slope of the sigmoid function, and C is the location of the midpoint of the sigmoid function in pixels [26].

Due to the geometry of the interfering ToF sensor, the sigmoid function parameters $\left[A,B,C\right]$ are determined based on the angle, θ, between the pixel (x,y) and the center pixel $\left(x_{c},y_{c}\right)$ of an IR light source as follows:

(2) $$r\left(\theta \right)=\left\{\begin{array}{c} \left[A_{1},B_{1},C_{1}\right],\;\;\;\;\;\;\alpha <\theta <\beta \;|\;\alpha +180{}^{\circ}<\theta <\beta +180{}^{\circ}\;\\ \left[A_{2},B_{2},C_{2}\right],\;\;\;\;\;\;\beta <\theta <\alpha +180{}^{\circ}\;|\;\beta +180{}^{\circ}<\theta <\alpha \end{array}\right.$$

where α and β demarcate different regions within the probability map.

**Figure 6:**
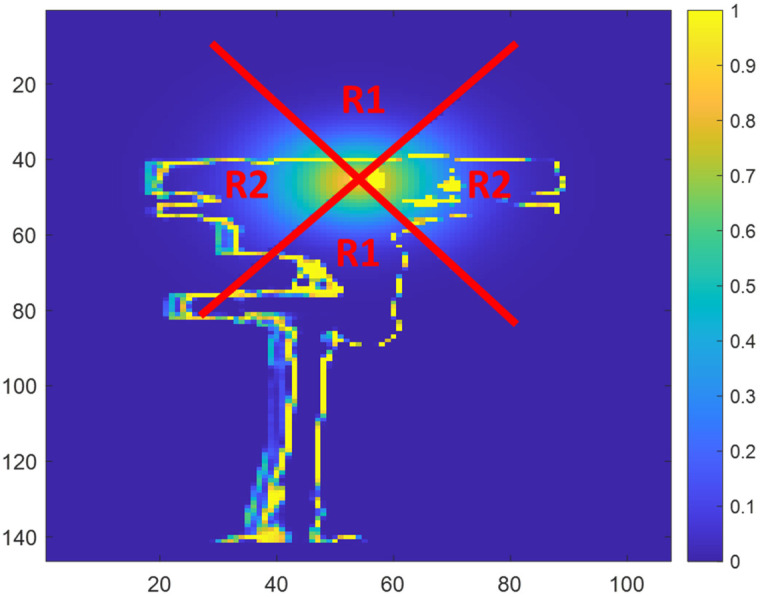
Regions used for determining the A, B, and C parameters for the sigmoid function. Region 1 (shown as R1) corresponds with radiuses along a vertical axis and Region 2 (shown as R2) corresponds with radiuses along a horizontal axis.

In our experiments the *A*, *B*, and *C* parameters of the sigmoid function are determined by first computing the probabilities of each pixel along radiuses projecting away from the IR light source. The empirical results suggest that these rates of decrease vary along a radial manner due to the geometry of the sensors. For simplicity, we only consider two different regions and choose different parameters for the sigmoid function in these two regions. Region 1 corresponds with radiuses between 45° and 135° and radiuses between 225° and 315°. Region 2 corresponds with radiuses between 315° and 45° and radiuses between 135° and 225°. Figure 6 illustrates Region 1 (shown as R1) and Region 2 (shown as R2). The probability values within each region were then averaged together based on the distance between the pixel and the center of the IR source. A non-linear least squares fitting of a sigmoid function is then performed on the average probability values within each region to determine the sigmoid function parameters A, B, and C for each region. A further constraint is that the sigmoid parameter A should be no larger than one, which corresponds with a maximum probability of 100% that a pixel is a zero-value pixel. This process results in a set of sigmoid parameters for each region. The determined sigmoid parameters A, B, and C depend on the relative physical locations of the primary ToF sensor and the interfering secondary ToF sensor with respect to each other. This relationship means that previously determined sigmoid parameters can be based on the physical locations of the primary ToF sensor and the interfering secondary ToF sensor.

For a given configuration of the ToF sensors, the sigmoid function parameters A, B, and C for each region are applied to the probability map to generate the pixel probabilities for a synthetic interference image. The pixel location of the center of the IR light source is first identified in a depth map containing the IR light source. The corresponding pixel for the center of the IR light source is then identified in the probability map. For each pixel in the probability map, a distance between the pixel and the pixel for the center of the IR light source, is determined. The sigmoid function from Equation (1) is then applied to determine a probability, $S\left(x,y\right)$, for the pixel based on the determined distance and the sigmoid parameters corresponding with the region where the pixel is located with respect to the IR light source. After determining probabilities for each pixel in the probability map, the probability of a pixel in the original depth map being a zero-value pixel when multicamera interference is present can be determined by identifying the probability associated with the corresponding pixel in the probability map.

### 2.3. Masking

**Figure 7:**
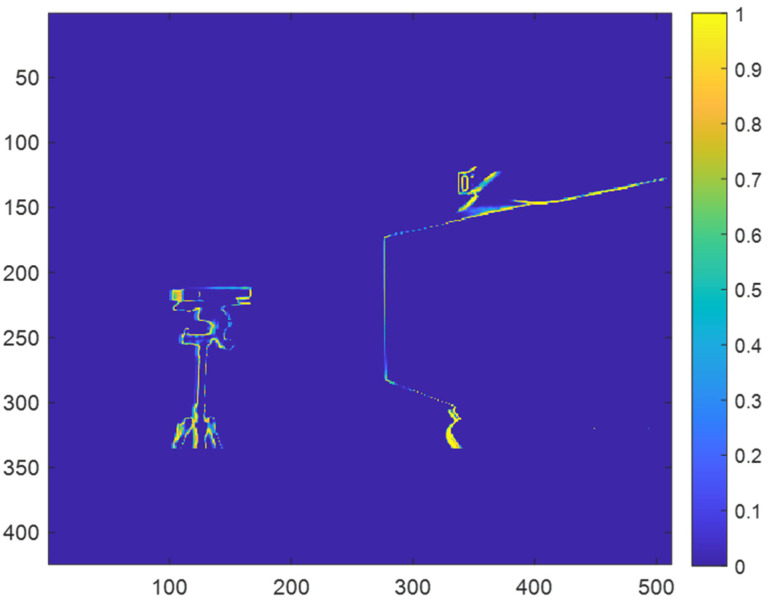
Example of prediction map for a depth map without multicamera interference.

After generating the probability map, the probability map can then be applied to a prediction map for a depth map without multicamera interference to synthetically generate direct multicamera interference in the depth map. Figure 7 shows an example of a prediction map for a depth map without multicamera interference. For each pixel, $\left(x,y\right)$, in the prediction map, ***I***, the new probability associated with the pixel when direct multicamera interference is present can be determined as follows.

(3) $$I\left(x,y\right)=\left\{\begin{array}{c} I\left(x,y\right)+S\left(x,y\right),\;\;\;\;\;\;\;\;\;\;\;\;\;\;\;\;\;\;\;\;\;\;\;\;\;\;\;\;\;I\left(x,y\right)+S\left(x,y\right)\leq 1\;\;\\ \;1,\;\;\;\;\;\;\;\;\;\;\;\;\;\;\;\;\;\;\;\;\;\;\;\;\;\;\;\;\;\;\;\;\;\;\;\;\;\;\;\;\;\;\;\;\;\;\;\;\;\;\;\;\;\;\;\;\;I\left(x,y\right)+S\left(x,y\right)>1 \end{array}\right.$$

**Figure 8:**
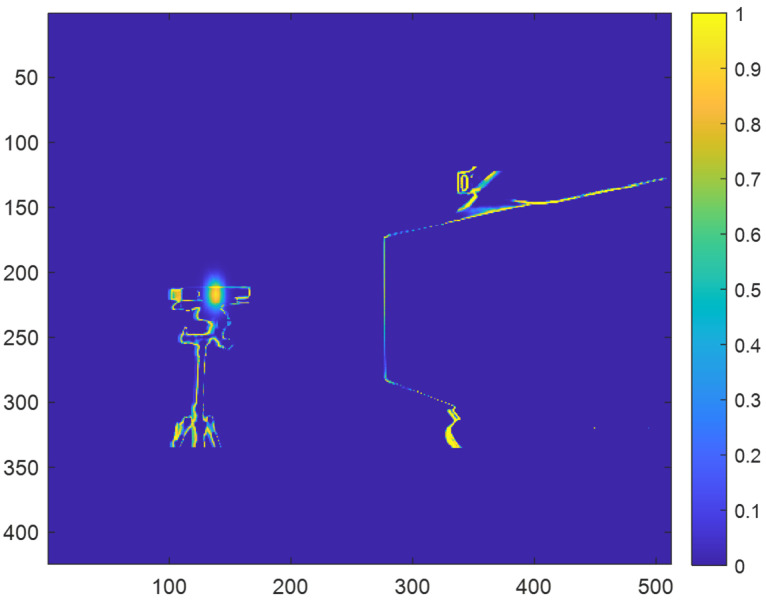
Example of a prediction for a depth map with synthetic direct multicamera interference after masking with the probability map.

Figure 8 shows an example of the prediction map for a depth map with synthetically generated direct multicamera interference after masking with the probability map.

### 2.4. Ray Tracing

In the case of indirect multicamera interference, a ray-tracing approach based on the geometry of the scene is used in conjunction with the probabilistic model discussed in Section 2.2 to predict zero-value pixels associated with indirect multicamera interference. This ray-tracing approach operates by virtually projecting and diffusing the IR light from an IR light source onto surfaces in a scene to predict the locations and probabilities of zero-value pixels that are associated with indirect interference.

**Figure 9:**
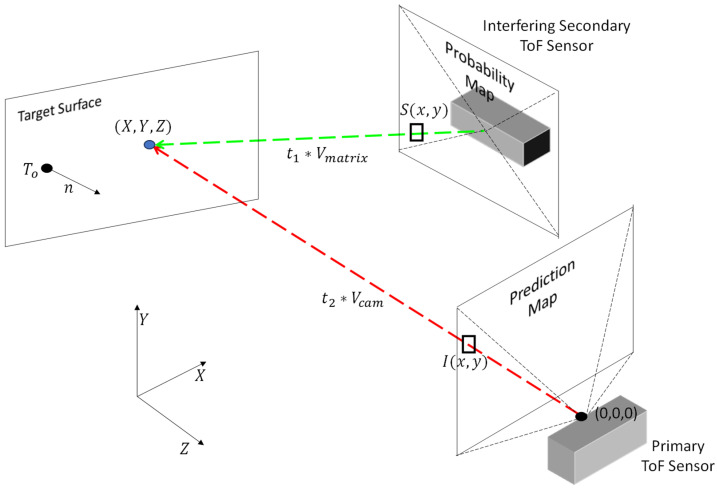
Perspective view of a primary ToF sensor configured to capture a depth map of a scene with a target surface and an interfering secondary ToF sensor that is also emitting IR light on the target surface.

Our ray-tracing approach is based on traditional vector-based 3D rendering techniques and uses a combination of forward ray tracing and reverse ray tracing [27,28,29,30,31]. Reverse ray tracing is first used to project the probabilities from the probability map onto surfaces within a scene. Forward ray tracing is then used to identify the probability values that are observed by each pixel in the primary ToF sensor. Figure 9 illustrates an example of forward ray tracing (shown in red) and reverse ray tracing (shown in green). We implemented our ray-tracing process, as described in detail below, using code that we developed in MATLAB. We developed our ray-tracing code based on the mathematical process described in references [28,29]. No other software or external libraries were used to implement this process.

To implement ray tracing, a set of vectors is first defined for each pixel in the prediction map which corresponds with a pixel in the primary ToF sensor [28]. Each pixel $\left(x,y\right)$ is first converted from the raster space to a normalized device coordinate space as follows.

(4) $$p_{NDC}\left(x\right)=\frac{\left(x+0.5\right)}{N}$$

(5) $$p_{NDC}\left(y\right)=\frac{\left(y+0.5\right)}{M}$$

In the normalized device space, the pixel values are normalized with respect to the dimensions of the image plane. The pixels are then converted from the normalized device space to a screen space as follows.

(6) $$p_{SS}\left(x\right)=2\;*\;p_{NDC}\left(x\right)-1$$

(7) $$p_{SS}\left(y\right)=1-2\;*\;p_{NDC}\left(y\right)$$

In the screen space, the origin is redefined as the center of the image plane. The pixels are then compensated for the aspect ratio, a, of the image plane as follows.

(8) $$a=\frac{N}{M}$$

(9) $$p_{CAR}\left(x\right)=a\;*\;p_{SS}\left(x\right)$$

(10) $$p_{CAR}\left(y\right)=p_{SS}\left(y\right)$$

The pixels are then adjusted to account for the FOV with respect to each axis of the image plane as follows:

(11) $$p_{FA}\left(x\right)=p_{CAR}\left(x\right)\;*\;\tan\left(\frac{f_{x}}{2}\right)$$

(12) $$p_{FA}\left(y\right)=p_{CAR}\left(y\right)\;*\;\tan\left(\frac{f_{y}}{2}\right)$$

where $f_{x}$ and $f_{y}$ correspond, respectively, to the FOV of the primary ToF sensor with respect to the image width N and the image height M. Vectors for each pixel in the image plane are then generated as follows:

(13) $$V_{camorigin}\left(x,y,z\right)=\left[0,0,0\right]$$

(14) $$V_{pixel}\left(x,y,z\right)=\left[p_{FA}\left(x\right),\;p_{FA}\left(y\right),-1\right]$$

(15) $$V_{cam}\left(x,y,z\right)=\left[V_{pixel}-V_{camorigin}\right]$$

where $V_{cam}\left(x,y,z\right)$ is a vector associated with a pixel $\left(x,y\right)$, $V_{camorigin}\left(x,y,z\right)$ is the origin and physical location of the primary ToF sensor within a scene, and $V_{pixel}\left(x,y,z\right)$ is the virtual physical location of each pixel of the image sensor within the scene.

A similar process is performed with respect to the interfering secondary ToF sensor and its associated probability map, $S\left(x,y\right)$ [28]. Vectors for each pixel in the probability map are generated as follows:

(16) $$V_{intorigin}\left(x,y,z\right)=\left[x_{int},y_{int},z_{int}\right]$$

(17) $$V_{matrix}\left(x,y,z\right)=\left[V_{intx},V_{inty},-1\right]$$

(18) $$V_{int}\left(x,y,z\right)=\left[V_{matrix}-V_{intorigin}\right]$$

where $V_{int}\left(x,y,z\right)$ is a vector associated with a pixel $\left(x,y\right)$, $V_{intorigin}\left(x,y,z\right)$ is the physical location of the interfering secondary ToF sensor within the scene, and $V_{matrix}\left(x,y,z\right)$ is the virtual physical location of each pixel of the probability map within the scene.

After generating vectors for the image plane and the probability map, the vectors associated with the probability map are then projected onto the scene [29]. For each vector in $V_{int}$, the parametric distance, $t_{1}$, between the interfering secondary ToF sensor and an intersection point on a target surface in the scene can be determined as follows:

(19) $$t_{1}=\frac{\left(T_{o}-V_{intorigin}\right)}{n\cdot V_{matrix}\left(x,\;y,\;z\right)}$$

where $T_{o}$ is any real-world point $\left(X,Y,Z\right)$ on the target surface and n is the normal of the target surface. The physical location of the intersection point, $g_{1}\left(X,Y,Z\right)$, in the scene is then determined as follows.

(20) $$g_{1}\left(X,Y,Z\right)=V_{intorigin}+V_{matrix}\left(x,y,z\right)\;*\;t_{1}$$

After projecting the vectors associated with the probability map, a similar process is performed with respect to the vectors of the primary ToF sensor [29]. For each vector in $V_{cam}$, the parametric distance, $t_{2}$, between the primary ToF sensor and an intersection point of the target surface in the scene can be computed as:

(21) $$t_{2}=\frac{\left(T_{o}-V_{camorigin}\right)}{n\cdot V_{pixel}\left(x,\;y,\;z\right)}$$

The physical location of the intersection point, $g_{2}\left(X,Y,Z\right)$, is then given by:

(22) $$g_{2}\left(X,Y,Z\right)=V_{camorigin}+V_{pixel}\left(x,y,z\right)\;*\;t_{2}$$

After identifying the intersection points for vectors in $V_{cam}$, the following process is then used to map a projected probability from the probability map to each pixel in the prediction map. For each intersection point, $g_{2}$, that is associated with a vector in $V_{cam}$, the nearest corresponding intersection point, $g_{1}$, that is associated with a vector $V_{int}$ is determined. After identifying the nearest corresponding intersection point $g_{1}$, the projected probability from the probability map can then be determined by identifying its corresponding vector $V_{int}$ and its associated probability value, $S\left(x,y\right)$, in the probability map.

To account for the specular reflection and diffusion from surfaces in the scene, the Phong model, which is a commonly used technique in 3D image rendering, is applied to the projected probability before mapping the probability value to the prediction map [30,31]. Using the Phong model, a modified probability value, $P\left(x,y\right)$, is determined as follows:

(23) $$P\left(x,y\right)=F_{D}+F_{S}$$

where $F_{D}$ corresponds with the diffusion component of the Phong model and $F_{S}$ corresponds with the specular reflection component of the Phong model. The diffusion component of the Phong model is determined as follows:

(24) $$F_{D}=k_{d}\;*\;\left(n\cdot V_{reflect}\right)\;*\;S\left(x,y\right)$$

where $k_{d}$ is a user-defined diffusion coefficient and $V_{reflect}$ is the reflection vector for the vector, $V_{cam}$, off of the target surface. $V_{reflect}$ is defined as follows.

(25) $$V_{reflect}=V_{cam}-2\;*\;\left(V_{cam}\cdot n\right)\;*\;n$$

The specular reflection component of the Phong model is determined as follows:

(26) $$F_{S}=k_{s}\;*\;{\left(V_{reflect}\cdot V_{cam}\right)}^{\varphi }\;*\;S\left(x,y\right)$$

where $k_{s}$ is a user-defined specular reflection coefficient and φ is a user-defined specular exponential coefficient. In our experiments, the values of $k_{d}$, $k_{s}$, and φ were selected based on the texture and reflectivity of our target surface [30].

After obtaining the modified probability value, $P\left(x,y\right)$, the pixel $\left(x,y\right)$ in the prediction map corresponding with the vector $V_{cam}$ is then masked with the modified probability value as follows.

(27) $$I\left(x,y\right)=\left\{\begin{array}{c} I\left(x,y\right)+P\left(x,y\right),\;\;\;\;\;\;I\left(x,y\right)+P\left(x,y\right)\leq 1\\ \;\;\;\;\;\;1,\;\;\;\;\;\;\;\;\;\;\;\;\;\;\;\;\;\;\;\;\;\;\;\;\;\;\;\;\;\;\;I\left(x,y\right)+P\left(x,y\right)>1\;\; \end{array}\right.$$

**Figure 10:**
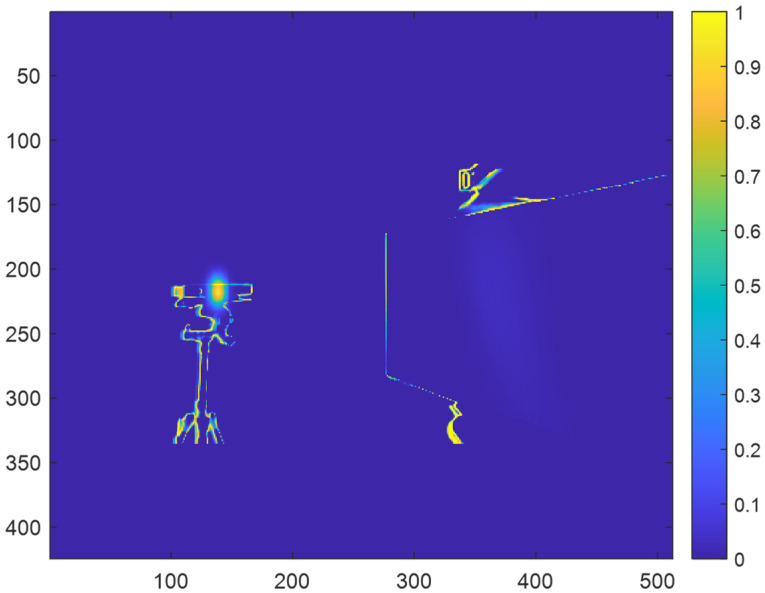
Example of prediction map for a depth map with synthetic direct multicamera interference and indirect multicamera interference after performing ray tracing.

Figure 10 shows an example of the prediction map for a depth map with synthetically generated direct multicamera interference and indirect multicamera interference after applying the modified probability values to the prediction map.

### 2.5. Generating Depth Map with Synthetic Multicamera Interference

The updated prediction map is then applied to the depth map without multicamera interference to synthetically generate multicamera interference in the depth map. For each pixel in the depth map, ***D***, the probability that a pixel is a zero-value pixel when multicamera interference is present can be applied as follows:

(28) $$D\left(x,y\right)=\left\{\begin{array}{c} D\left(x,y\right),\;\;\;\;\;\;\;\;\;\;\;\;\;\;\;\;\;\;\;\;\;\;\;I\left(x,y\right)<t\;\;\\ \;0,\;\;\;\;\;\;\;\;\;\;\;\;\;\;\;\;\;\;\;\;\;\;\;\;\;\;\;\;\;\;\;\;I\left(x,y\right)\geq t \end{array}\right.$$

where t is a user-defined probability threshold value.

## 3. Experiments

### 3.1. Hardware Configuration

In our experiments, we used Kinect v2 sensors [32] as both the primary data collecting ToF sensor and the interfering source. The Kinect v2 ToF sensor has a resolution of 512 × 424 pixels and a framerate of 30 frames per second [33]. For capturing depth images, we used the OpenKinect libraries [34]. In our experiments, we disabled both the Bilateral filter and the Edge-aware filter within the OpenKinect libraries to ensure raw depth information is captured [35]. In order to implement our multicamera interference model and process, we used MATLAB R2020b software [36].

For each experiment configuration, the primary ToF sensor collected depth images of the scene in front of the primary ToF sensor over a thirty-minute time period. During this data collection period, an average of 13,634 depths images were collected. This process was repeated multiple times for each combination of position angle and distance. The test data for our experiments was then generated by computing the probabilities for each pixel being a zero-value pixel within each set of depth images for each combination of position angle and distance.

**Figure 11:**
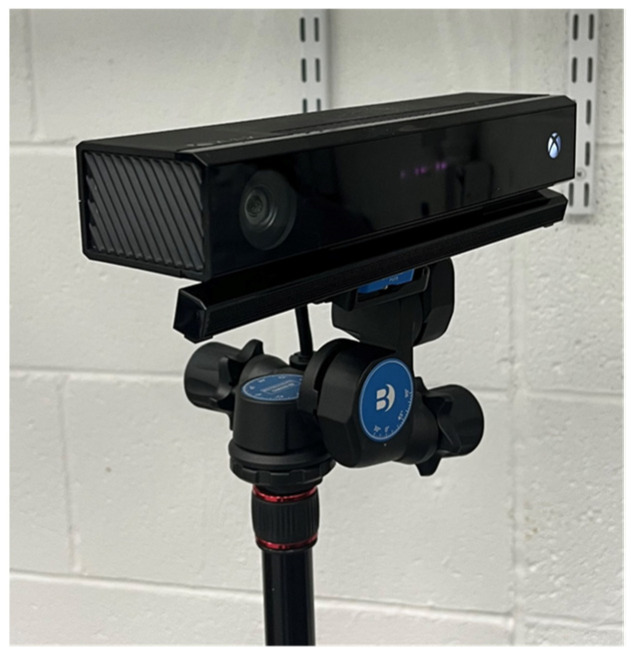
Interfering secondary ToF sensor mounted to a tripod using the Benro 3-Way Geared Head.

The interfering secondary ToF sensor is mounted on a tripod using a Benro 3-Way Geared Head [37], which allows for precise control of the yaw, pitch, and roll. The configuration of the interfering secondary ToF sensor is shown in Figure 11. We used a dual-axis digital protractor [38] to ensure the interfering secondary ToF sensor is level with the primary ToF sensor. We also used laser range finders to measure the distances between the interfering secondary ToF sensor, the primary ToF sensor, and the reflected surface.

**Figure 12:**
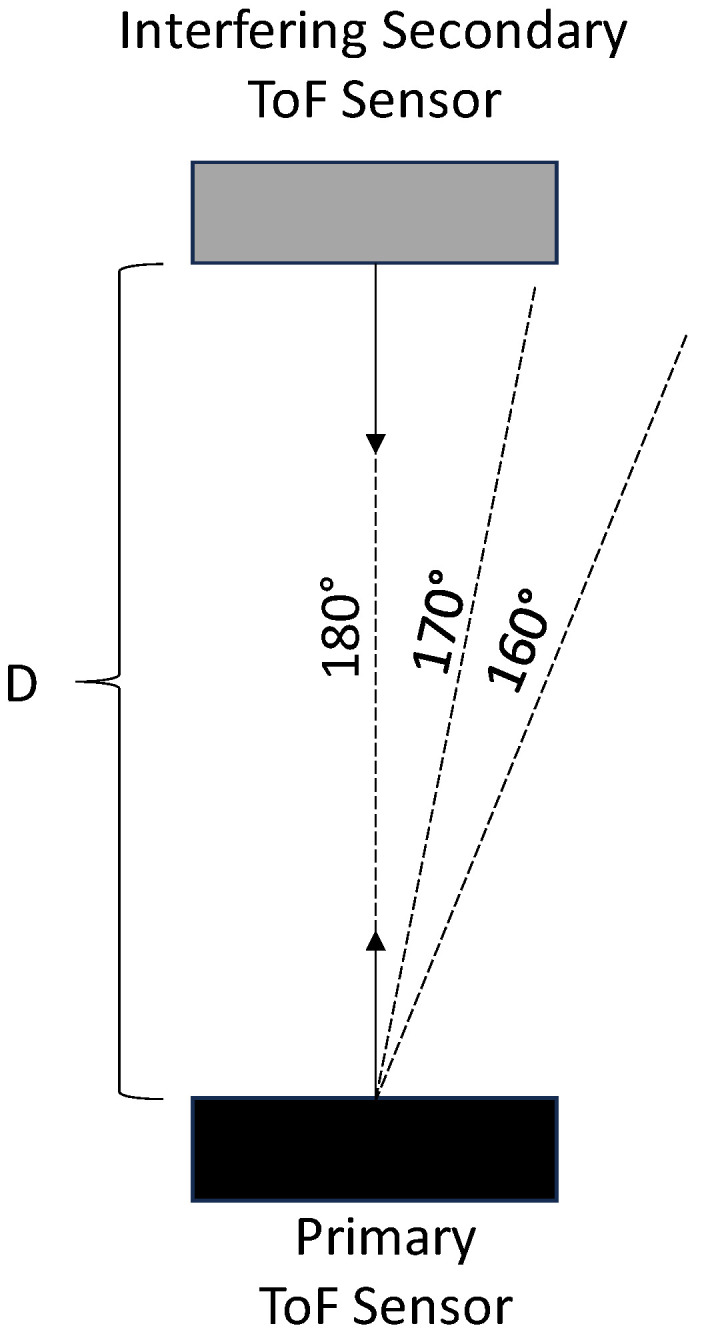
Top view of hardware configuration for direct interference experiments.

Figure 12 illustrates a top view of the hardware configuration that was used for our direct multicamera interference experiments. In this configuration, the primary ToF sensor and the interfering secondary ToF sensor are positioned such that the IR light from the interfering secondary ToF sensor is emitted directly towards the primary ToF sensor. For these experiments, the interfering secondary ToF sensor was positioned facing angles of 180°, 170°, and 160° with respect to the image plane of the primary ToF sensor. At each of these position angles, the interfering secondary ToF sensor was positioned at distances (shown as distance D) of 1.0 m, 1.2 m, 1.4 m, and 1.6 m away from the primary ToF sensor.

**Figure 13:**
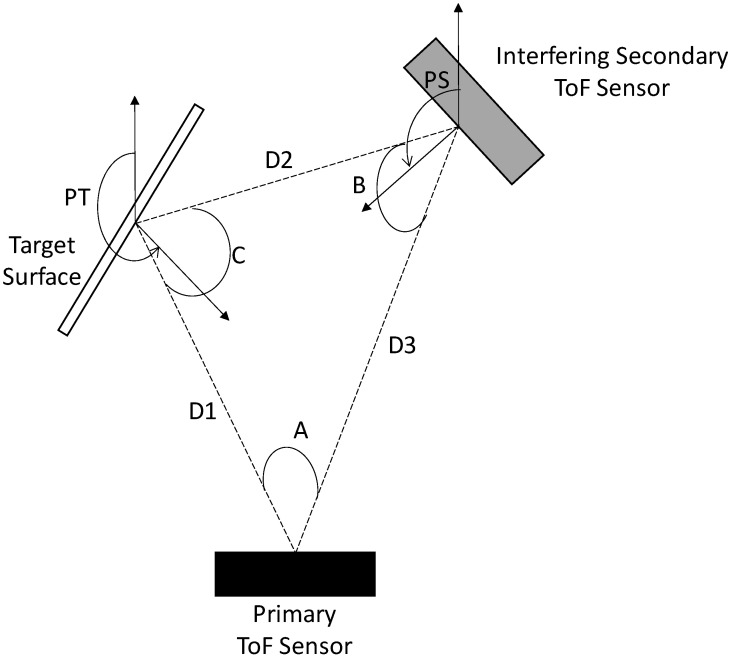
Top view of hardware configuration for combined direct and indirect interference experiments.

Figure 13 illustrates a top view of the hardware configuration that was used for our combined direct and indirect multicamera interference experiments. In this configuration, the primary ToF sensor and the interfering secondary ToF sensor are positioned such that a portion of the IR light from the interfering secondary ToF sensor is emitted directly towards the primary ToF sensor. In this configuration, two ToF sensors are also positioned such that another portion of the IR light from the interfering secondary ToF sensor is reflected off of a target surface before being captured by the primary ToF sensor. In this experiment, the target surface was a matte white foamboard. Both the interfering secondary ToF sensor and the target surface were within the FOV of the primary ToF sensor.

For these experiments, the physical locations of the primary ToF sensor, the interfering secondary ToF sensor, and the target surface remained fixed. The distance between the primary ToF sensor and the target surface (shown as D1) was 1.15m. The distance between the primary ToF sensor and the interfering secondary ToF sensor (shown as D3) was 1.6m. The distance between the target surface and the interfering secondary ToF sensor (shown as D2) was 1.03m. The primary ToF sensor was configured with a position angle (shown as A) of 40° between the interfering secondary ToF sensor and the target surface. The interfering secondary ToF sensor was configured with a position angle (shown as B) of 46° between the primary ToF sensor and the target surface. The target surface was configured with a position angle (shown as C) of 94° between the primary ToF sensor and the interfering secondary ToF sensor. For these experiments, the interfering secondary ToF sensor was configured with pivot angles (shown as PS) of 145°, 140°, 135°, and 130° degrees with respect to the image plane of the primary ToF sensor. The pivot angle of the target surface (shown as PT) remained fixed at 205° with respect to the image plane of the primary ToF sensor. These angles were selected to ensure that both direct and indirect interference can be observed in all cases to ensure efficiency in our experimental process.

### 3.2. Performance Metrics

To evaluate the performance of the zero-value pixel predictions from the probabilistic model described in Section 2.2, we used the following commonly used metrics for comparing images [39,40]: the root mean square error (RMSE) [41], peak signal-to-noise ratio (PSNR) [42,43], and structural similarity index measure (SSIM) [44,45]. These metrics were used to compare the prediction maps with synthetic multicamera interference and the prediction maps with real multicamera interference.

Our process for evaluating synthetic multicamera interference performance involved first capturing a series of depth maps with multicamera interference where the position and orientation of the primary ToF sensor and the interfering secondary ToF sensor remained fixed during the data collection. In our experiments, the primary ToF sensor collected the series of depth maps over a thirty-minute time period to ensure statistical consistency. Our experiments showed that a thirty-minute time period was a sufficient amount of time for the Kinect sensor to cycle though one period of its frequency-hopping patterns.

We then generated a prediction map with real multicamera interference where each pixel was associated with a probability of being a zero-value pixel based on the number of instances that the pixel was a zero-value pixel within the collected data sample. For example, a pixel that had a zero-value for the entire data sample was associated with a probability of 100%, a pixel that had a zero-value for half of the data sample was associated with a probability of 50%, and so on.

Next, we generated a prediction map with synthetic multicamera interference that corresponded with the position and orientation of the primary ToF sensor and the interfering secondary ToF sensor. Similar to the prediction map with real multicamera interference, the pixels in the prediction map with synthetic multicamera interference were configured such that each pixel was associated with a probability of being a zero-value pixel based on our probabilistic model and framework. The prediction map with real multicamera interference and the prediction map with synthetic multicamera interference were then directly compared pixel-by-pixel to determine the RMSE, PSNR, and SSIM.

### 3.3. Direct Interference Experimental Results

**Figure 14:**
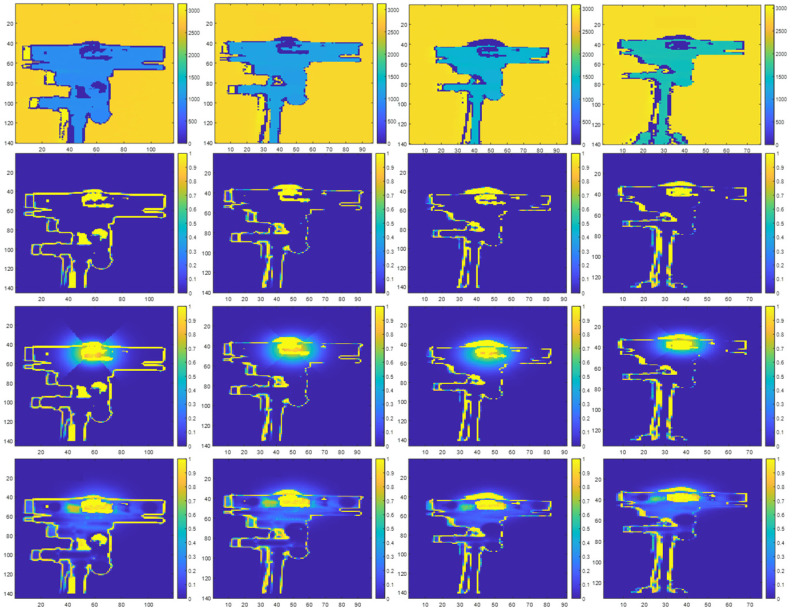
Interfering ToF sensor with a position angle of 180° with respect to the primary ToF sensor initial depth map in millimeter units (**first row**), prediction map without direct multicamera interference (**second row**), prediction map with synthetic direct multicamera interference (**third row**), and prediction map with real direct multicamera interference (**fourth row**) at distances of 1.0 m, 1.2 m, 1.4 m, and 1.6 m (**left**–**right**) between the interfering ToF sensor and the primary ToF sensor.

Figure 14 illustrates examples of prediction maps from our direct interference experiments that show the locations and probabilities of zero-value pixels. Figure 14 corresponds to the results when the interfering secondary ToF sensor is located with a position angle of 180° with respect to the primary ToF sensor. In this configuration, the interfering secondary ToF sensor and the primary ToF sensor are aligned and directly facing each other.

In each figure, the first row shows prediction maps of the interfering secondary ToF sensor without direct multicamera interference, the middle row shows prediction maps with synthetic direct multicamera interference, and the bottom row shows prediction maps with real direct multicamera interference at different distances between the interfering secondary ToF sensor and the primary ToF sensor.

Table 2 shows the sigmoid parameter values that were determined based on our probabilistic model for the direct interference experiments. Axis 1 corresponds with a vertical axis corresponding with Region 1 and Axis 2 corresponds with a horizontal axis corresponding with Region 2. Parameters A, B, and C correspond to the sigmoid parameters discussed in Section 2.2.

**Figure 15:**
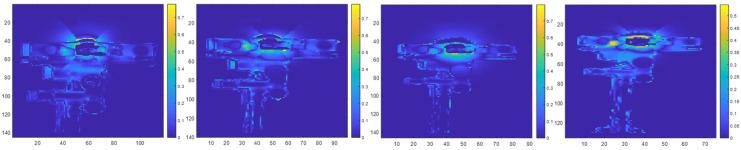
Direct multicamera interference error maps showing absolute difference between real multicamera interference and synthetic multicamera interference prediction maps at distances of 1.0 m, 1.2 m, 1.4 m, and 1.6 m (**left**–**right**).

Our results show that at each position angle, the midpoint of the sigmoid function (i.e., parameter C) generally changes monotonically and is inversely proportional to the distance between the primary ToF sensor and the interfering ToF sensor. This behavior translates to the size (e.g., radius) of the direct multicamera interference increasing when the distance between the primary ToF sensor and the interfering ToF sensor is reduced and decreasing when the distance between the primary ToF sensor and the interfering ToF sensor increases. Due to the physical configuration of the Kinect V2 sensor’s IR emitters, which are positioned horizontally along the body of the Kinect, at a given position angle and distance, the midpoint of the sigmoid function tends to be slightly larger along the horizontal directions (i.e., Axis 2) compared to the vertical directions (i.e., Axis 1). In our experiments, the midpoint of the sigmoid function generally ranged from 6.8 to 10 pixels in the vertical directions and ranged from 8.6 to 14.19 pixels in the horizontal directions. The slope of the sigmoid function (i.e., parameter B) typically varied between −0.19 and −0.36 with an average value of −0.2564 across all position angles and distances. The peak value of the sigmoid function (i.e., parameter A) was one for all position angles and distances. A peak value of one corresponds with a probability of 100% for a zero-value pixel being present in a depth map. As shown in Figure 14, these peak values typically correspond with the center of the IR source of the interfering ToF sensor. Figure 15 shows the corresponding direct multicamera interference error maps for the results shown in Figure 14.

Table 3 shows the performance of the proposed synthetically generated direct multicamera interference process. For each position angle and distance, we compared the prediction maps of the interfering ToF sensor with synthetic direct multicamera interference and real direct multicamera interference. Table 3 shows that the proposed framework for synthetically generating direct multicamera interference achieves an average RMSE of 0.0625, an average PSNR of 24.1277 dB, and an average SSIM of 0.9007 for position angles between 180 and 160°. Our results show that a similar level of performance can be achieved across all tested position angles and distances. In general, our results show that the locations and probabilities associated with zero-value pixels can be accurately predicted for direct multicamera interference, which translates to the accurate synthetic representation of direct multicamera interference.

### 3.4. Indirect Interference Experiment Results

**Figure 16:**
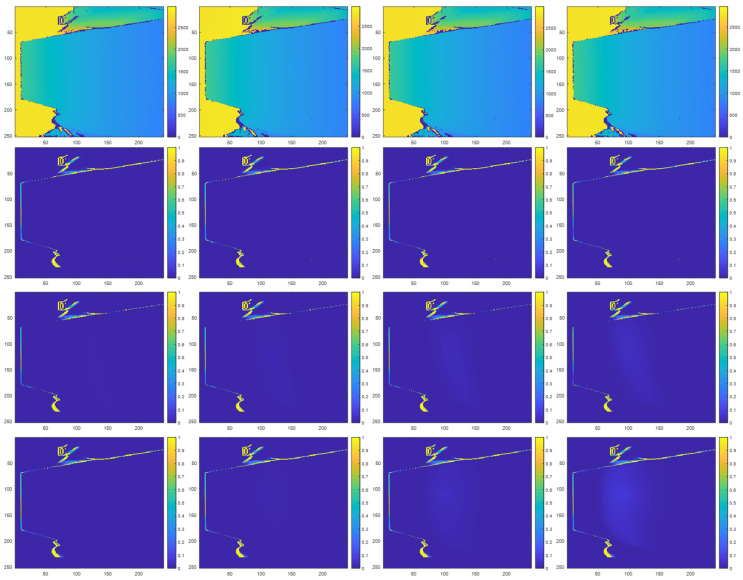
Indirect multicamera interference initial depth map in millimeter units (**first row**), prediction map without multicamera interference (**second row**), prediction map with synthetic multicamera interference (**third row**), and prediction map with real multicamera interference (**fourth row**) at pivot angles of 15°, 20°, 25°, and 30° (**left**–**right**) for the interfering ToF sensor with respect to the image plane of the primary ToF sensor.

Figure 16 illustrates prediction maps from our indirect interference experiments that show the locations and probabilities of zero-value pixels. Figure 16 corresponds to the results when the interfering ToF sensor remains at a fixed position and sweeps its pivot angle with respect to a target surface. In Figure 16, the first row shows prediction maps of the target without indirect multicamera interference, the middle row shows prediction maps with synthetic indirect multicamera interference, and the bottom row shows prediction maps with real indirect multicamera interference at different pivot angles between the interfering secondary ToF sensor and the target surface.

**Figure 17:**
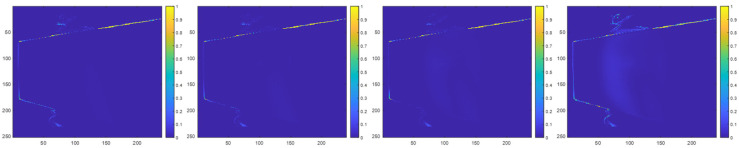
Indirect multicamera interference error maps showing absolute difference between real multicamera interference and synthetic multicamera interference prediction maps at pivot angles of 15°, 20°, 25°, and 30° (**left**–**right**).

Our results show that through ray tracing, the effect of the zero-value pixels associated with direct multicamera interference can be successfully projected and diffused onto other surfaces within a scene to mimic the behavior of indirect multicamera interference. Figure 17 shows the corresponding indirect multicamera interference error maps for the results shown in Figure 16.

Table 4 shows the performance of the proposed synthetically generated indirect multicamera interference process. For each pivot angle, we compare the prediction maps of the target with synthetic indirect multicamera interference and real indirect multicamera interference. Table 4 shows that the proposed framework for synthetically generating indirect multicamera interference achieves an average RMSE of 0.0312, an average PSNR of 26.2280 dB, and an average SSIM of 0.9064 for pivot angles between 15 and 30°. Similar to the case of direct multicamera interference, our results show that the locations and probabilities associated with zero-value pixels can be accurately predicted for indirect multicamera interference, which translates to the accurate synthetic representation of indirect multicamera interference, and ultimately combined direct and indirect multicamera interference.

### 3.5. Combined Direct and Indirect Interference Experiment Results

**Figure 18:**
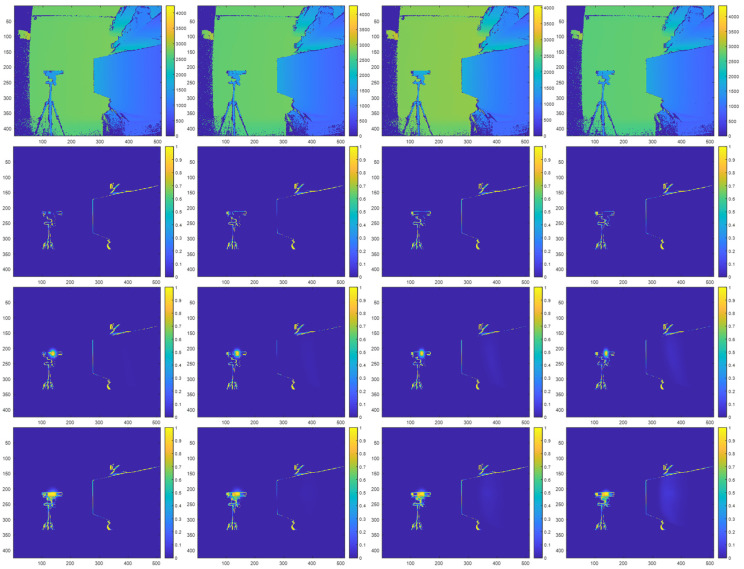
Combined direct and indirect multicamera interference configuration initial depth map in millimeter units (**first row**), prediction maps without multicamera interference (**second row**), prediction maps with synthetic multicamera interference (**third row**), and prediction maps with real multicamera interference (**fourth row**) at pivot angles of 15°, 20°, 25°, and 30° (**left**–**right**) for the interfering ToF sensor with respect to the image plane of the primary ToF sensor.

**Figure 19:**
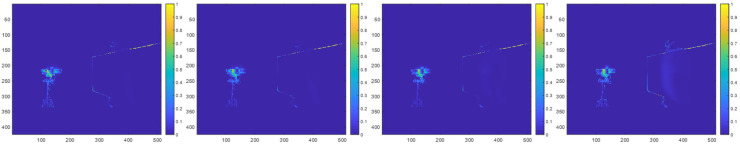
Combined direct and indirect multicamera interference error maps showing absolute difference between real multicamera interference and synthetic multicamera interference prediction maps at distances of 15°, 20°, 25°, and 30° (**left**–**right**).

Figure 18 illustrates prediction maps from our combined direct and indirect interference experiments that show the locations and probabilities of zero-value pixels. Figure 16 combines the individual components of direct multicamera interference and indirect multicamera interference from Figure 14 and Figure 16 above to illustrate a composite prediction map that includes both direct and indirect multicamera interference. Similar to above, Figure 18 corresponds to the results when the interfering ToF sensor remains at a fixed position and sweeps its pivot angle with respect to a target surface. In Figure 18, the first row shows prediction maps of the interfering ToF sensor and the target without multicamera interference, the middle row shows prediction maps with synthetic multicamera interference, and the bottom row shows prediction maps with real multicamera interference at different pivot angles between the interfering secondary ToF sensor and the target surface. Figure 19 shows the corresponding combined direct and indirect multicamera interference error maps for the results shown in Figure 18.

### 3.6. Non-Zero-Value Pixel Experiment Results

**Figure 20:**
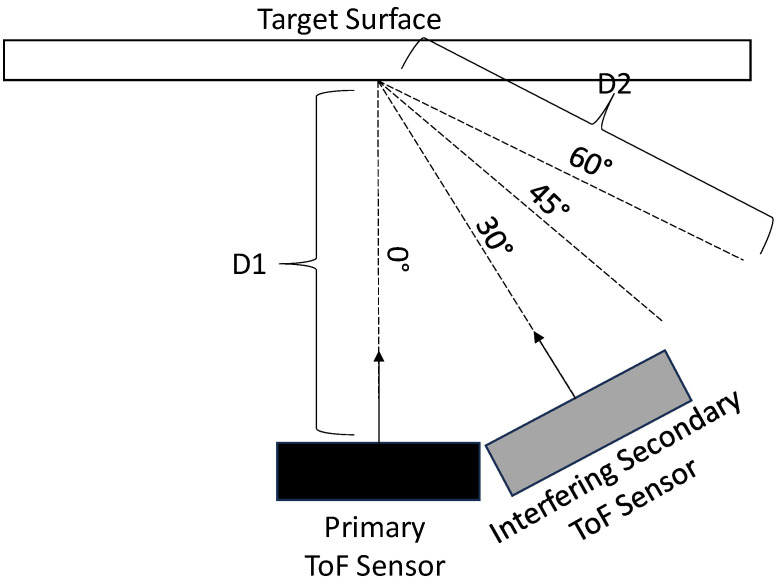
Top view of the hardware configuration for non-zero-value pixel error experiments.

Figure 20 illustrates a top view of the hardware configuration that was used in our experiments for determining the amount of error that is experienced by non-zero-value pixels due to multicamera interference. In these experiments, the primary ToF sensor was positioned fronto-parallel with a target planar surface at a fixed distance of 1.0 m (shown as D1). The interfering secondary ToF sensor was configured such that the IR light from the interfering secondary ToF sensor is reflected off of the target surface before being captured by the primary ToF sensor. For these experiments, the interfering secondary ToF sensor was positioned at angles of 30°, 45°, and 60° with respect to the image plane of the primary ToF sensor. At each of these position angles, the interfering secondary ToF sensor was positioned at distances (shown as distance D2) of 0.8 m, 1.0 m, 1.2 m, and 1.4 m away from the target surface.

Table 5 shows experimental results for the amount of error that is experienced by non-zero-value pixels due to multicamera interference. Table 5 shows that non-zero-value pixels experienced an average error of 0.52 mm across angles between 30 and 60° and distances between 0.8 and 1.4 m between the interference source and the target surface.

## 4. Conclusions

In this work, we presented a framework for synthetically generating multicamera interference in depth maps that mirrors the behavior of real multicamera interference that is observed using a ToF sensor. This work introduced a framework and probabilistic model that can predict the locations and probabilities of zero-value pixels that are present when multicamera interference is present based on the physical locations of ToF sensors with respect to each other. This work also introduced a process for synthetically generating both direct multicamera interference and indirect multicamera interference.

Our results show that direct multicamera interference increases when the position angle between the primary ToF sensor and the interfering secondary ToF sensor approaches 180° (i.e., the ToF sensors are directly facing each other), and when the distance between the ToF sensors decreases. Conversely, direct multicamera interference decreases when the position angle between the primary ToF sensor and the interfering secondary ToF sensor approaches 90° (i.e., the ToF sensors are perpendicular with each other), and when the distance between the ToF sensors increases. For indirect multicamera interference, multicamera interference increases when the position angle between the primary ToF sensor and the interfering secondary ToF sensor approaches 0° (i.e., the ToF sensors are parallel with each other), and when the distance between the interfering secondary ToF sensor and a target surface decreases. Indirect multicamera interference decreases when the position angle between the primary ToF sensor and the interfering secondary ToF sensor approaches 90° and when the distance between the interfering secondary ToF sensor and a target surface increases.

The work can also be extended to include interference from multiple ToF sensors and from non-planar reflectors. Future works can use our framework and model as the basis for developing techniques and algorithms for mitigating the effects of multicamera interference in depth maps that are observed using a ToF sensor. Future works may also consider ways to predict sigmoid parameters to be used in other geometrical configurations.

## Figures and Tables

**Table 1:** Frequently used notations in this paper.

Symbol	Description
D	The depth map in 2D matrix of pixels with dimensions M×N
I	The prediction map in 2D matrix of pixels with dimensions M×N
P	The probability map in 2D matrix of pixels with dimensions M×N
$S\left(x,y\right)$	The sigmoid function probability of a pixel being a zero-value pixel
A	The peak probability value for the sigmoid function
B	The slope of the sigmoid function
C	The location of the midpoint of the sigmoid function in pixels
$V_{camorigin}\left(x,y,z\right)$	The origin and physical location of the primary ToF sensor
$V_{pixel}\left(x,y,z\right)$	The virtual physical location of a pixel of the primary ToF sensor
$V_{cam}\left(x,y,z\right)$	The vector associated with a pixel of the ToF sensor
$V_{intorigin}\left(x,y,z\right)$	The physical location of the interfering secondary ToF sensor
$V_{matrix}\left(x,y,z\right)$	The virtual physical location of a pixel of the interfering secondary ToF sensor
$V_{int}\left(x,y,z\right)$	The vector associated with a pixel of the interfering secondary ToF sensor
$t_{1}$	The parametric distance between the interfering secondary ToF sensor and an intersection point on a target surface
$g_{1}\left(X,Y,Z\right)$	The physical location of an intersection point on a target surface for a pixel vector from the interfering secondary ToF sensor
$t_{2}$	The parametric distance between the primary ToF sensor and an intersection point on a target surface
$g_{2}\left(X,Y,Z\right)$	The physical location of an intersection point on a target surface for a pixel vector from the primary ToF sensor
$P\left(x,y\right)$	The modified probability value for a pixel from the Phong model for specular reflection and diffusion
$k_{d},\;k_{s},\varphi$	The Phong model diffusion, specular reflection, and specular exponential coefficients
$V_{reflect}$	$\mathrm{The}\;\mathrm{reflection}\;\mathrm{vector}\;\mathrm{for}\;a\;\mathrm{vector},\;V_{cam}$, associated with the primary ToF sensor
$T_{o}$	The physical location of a point on a target surface
n	The surface normal of a target surface

**Table 2:** Sigmoid parameter values for direct multicamera interference.

Position Angle = 180°	Axis 1			Axis 2		
Distance:	A	B	C	A	B	C
1.0 m	1	−0.3516	10.020	1	−0.1912	13.870
1.2 m	1	−0.2312	9.382	1	−0.2217	11.640
1.4 m	1	−0.1931	9.003	1	−0.2249	9.961
1.6 m	1	−0.3300	7.328	1	−0.2912	8.608
Position Angle = 170°	Axis 1			Axis 2		
Distance:	A	B	C	A	B	C
1.0 m	1	−0.2504	9.672	1	−0.2004	12.170
1.2 m	1	−0.2489	8.188	1	−0.2812	10.410
1.4 m	1	−0.3560	7.063	1	−0.2869	9.596
1.6 m	1	−0.3691	6.824	1	−0.2548	9.378
Position Angle = 160°	Axis 1			Axis 2		
Distance:	A	B	C	A	B	C
1.0 m	1	−0.2093	10.010	1	−0.2081	14.190
1.2 m	1	−0.2560	8.302	1	−0.2099	12.230
1.4 m	1	−0.3097	7.346	1	−0.2649	10.370
1.6 m	1	−0.2288	7.103	1	−0.1845	10.700

**Table 3:** Synthetic direct multicamera interference performance.

Position Angle = 180°			
Distance:	RMSE	PSNR (dB)	SSIM
1.0 m	0.0668	23.4984	0.8733
1.2 m	0.0695	23.1541	0.9037
1.4 m	0.0637	23.9189	0.8920
1.6 m	0.0584	24.6660	0.9286
Position Angle = 170°			
Distance:			
1.0 m	0.0564	24.9814	0.9079
1.2 m	0.0534	25.4517	0.9084
1.4 m	0.0547	25.2334	0.9147
1.6 m	0.0672	23.4590	0.9084
Position Angle = 160°			
Distance:			
1.0 m	0.0582	24.7000	0.9044
1.2 m	0.0618	24.1834	0.8964
1.4 m	0.0616	24.2029	0.9061
1.6 m	0.0787	22.0837	0.8642

**Table 4:** Synthetic indirect multicamera interference performance.

Pivot Angle:	RMSE	PSNR (dB)	SSIM
15°	0.0496	26.0831	0.9440
20°	0.0397	28.0190	0.9456
25°	0.0227	32.8678	0.9462
30°	0.0127	17.9422	0.7897

**Table 5:** Non-zero-value pixel depth errors due to multicamera interference.

Position Angle = 30°				
Distance:	0.8 m	1.0 m	1.2 m	1.4 m
Error:	1.50 mm	1.10 mm	0.09 mm	0.08 mm
Position Angle = 45°				
Distance:	0.8 m	1.0 m	1.2 m	1.4 m
Error:	0.09 mm	0.06 mm	0.06 mm	0.04 mm
Position Angle = 60°				
Distance:	0.8 m	1.0 m	1.2 m	1.4 m
Error:	3.00 mm	0.07 mm	0.06 mm	0.05 mm

## Data Availability

Not applicable.
